# A High Distribution of Resistant Pathogens among Critically Ill Neonates from Secondary Referral Hospital of Indonesia

**DOI:** 10.4314/ejhs.v31i3.6

**Published:** 2021-05

**Authors:** Cucunawangsih Cucunawangsih, Paulus Mario Christopher, Nicolaski Lumbuun

**Affiliations:** 1 Microbiology Department, Faculty of Medicine, Pelita Harapan University, Jendral Sudirman Boulevard, Lippo Karawaci, Tangerang, Banten, Indonesia 15811; 2 Faculty of Medicine, Pelita Harapan University, Jendral Sudirman Boulevard, Lippo Karawaci, Tangerang, Banten, Indonesia 15811; 3 Pharmacology Department, Faculty of Medicine, Pelita Harapan University, Jendral Sudirman Boulevard, Lippo Karawaci, Tangerang, Banten, Indonesia 15811

**Keywords:** Neonates, multidrug-resistance pathogens, Gram negative bacteria, susceptibility

## Abstract

**Background:**

The spread of resistant pathogens among critically ill neonates has increased in recent years. Therefore, information about the antimicrobial profile and its susceptibility over time helps to select the most appropriate therapy. The study assesses the distribution of resistant pathogens and its susceptibility among neonates' patients.

**Methods:**

Eight hundred and eight suspected neonatal infected from January 2011 to December 2019 were recruited anonymously in our retrospective, observational analysis. The study was conducted in the secondary-care level NICU which located on the western border of Jakarta, Indonesia. The MDROs definition was define by Centre for Disease Prevention and Control (CDC) criteria and standardized international terminology. Microbial identification and susceptibility testing were carried out following standard protocols.

**Results:**

Culture positivity was found in 132 (16.3%) with dominating MDR-Gram negative bacteria 47 (61.8%). The most common pathogens were extended-spectrum β-lactamase and multidrug-resistant Acinetobacter 18 (38.3%), respectively. There were coagulase negative staphylococci 29 (38.2%) among MDROs. Most of the Gram negative bacteria were highly susceptible to the combination of cefoperazone/sulbactam (79.6%), amikacin (88.7%), and tigecycline (77.1%). Staphylococcus aureus had a good susceptibility to almost all classes' antibiotics. Candida isolates showed 100.0% susceptibility to all antifungal classes.

**Conclusions:**

Our study highlighted the microbial profile along with its susceptibility among neonatal patients that able to provide necessary information for antimicrobial guidelines and policies for effective infectious case management.

## Introduction

There is a widespread rapid emergence of resistant pathogens in the world. Globally, multidrug-resistance (MDR) pathogens have been declared a significant health threat for the community and healthcare facilities (1,2). Due to these pathogens in highly vulnerable patients with excessive use of invasive procedures and high consumption of newer antibiotics, infection contributes to increase mortality and morbidity, including prolonged hospitalization by 6.4 to 12.7 days and worsen clinical outcomes (1,3).

Increased circulating MDR pathogens, significantly Gram-negative bacteria (GNB), have been reported, especially from a critical care unit such as the neonatal intensive care unit (NICU). The NICU is a predominantly unavoidable exposure to various risk factors on critically ill subjects (3–6). However, the incidence of resistant pathogens in NICU is challenging to overcome, accompanied by the complexity of their resistance determinants between hospital and community settings. Many risk factors and conditions underlie, including pre-term age, previous broad-spectrum antibiotics, comorbid renal disease, and exposure to invasive devices (3,5).

Many studies related to healthcareassociated neonatal infection have been carried out worldwide to recognize the antimicrobial resistance patterns. The ecological pathogenic profiles were not equally year-by-year due to inappropriate and over-use of antimicrobials worldwide (1,7–8). Neonatal infections need to be treated promptly by empirical antimicrobial therapy in order to reduce mortality. The option for antimicrobial therapy is based on the epidemiological privilege of the pathogen patterns and their susceptibility in each region (8–10). Precise and timely evidence on the local and national antimicrobial profile is essential for good clinical practice and comparisons (11). Nevertheless, the available data for the microbial profile and antibiotic susceptibility were scanty, especially in Indonesia. So, we proposed the pathogens profile and their susceptibility, including multi-resistance bacteria in a NICU setting, to cater to the need for guidance on selecting the appropriate empirical antibiotic therapy at the local level as well as similar facilities for a benchmark.

## Methods

The study design was a retrospective observational analysis covering 808 suspected neonatal infection cases in Siloam Teaching Hospital in conjunction with the Department of Microbiology, Faculty of Medicine, Pelita Harapan University, Tangerang (located on the western border of Jakarta). We collected the data of patients treated at the NICU from January 2011 to December 2019 from the laboratory database. Therefore, clinical data could not be included, but only data on the source of infection and pathogens were available. Data were anonymized before analysis by de-identifying patients. The review board of the Faculty of Medicine, Pelita Harapan University, approved ethical clearance.

**Definition for multidrug-resistant pathogens**: MDR isolates were determined based on the criteria according to the European Centre for Disease Prevention and Control (ECDC) and the Centers for Disease Control and Prevention (CDC). An isolate was confirmed as a multidrug pathogen if it proofs to be non-susceptible to at least one drug in ≥3 antimicrobial classes being testing (12–13). The criterion for defining Methicillin-Resistant *Staphylococcus aureus* (MRSA) was determined using cefoxitinresistant or oxacillin-resistant as a marker. Extended-Spectrum β-lactamase (ESBL) producing isolates were determined by CLSI confirmatory testing (14). MDR-Acinetobacter (MDR-Ab) was defined as any *Acinetobacter spp*. which showed non-susceptible to ≥1 drug in at least three of the antimicrobial classes (β-lactams and β-lactamase inhibitor combinations; sulbactam; antipseudomonal cephalosporins; carbapenems; aminoglycosides; and fluoroquinolones).

**Specimen processing:** Specimen quality and direct Gram staining were performed prior to the inoculation. The sputum, urine, cerebrospinal fluid (CSF), feces, and pus were simultaneously inoculated onto 5% blood agar plates; selective and differential medium such as MacConkey agar for GNB, mannitol salt agar for Gram-positive bacteria (GPB), and CHROMagar™ for candida were used. Blood cultures were performed following the protocol from BD BACTEC™ automated blood culture systems. All cultures were incubated aerobically at 37°C for 18–24 hours, and the negative cultures were incubated for up to five days for bacteria and nine days for candida/other fungi before reported as negative.

**Pathogen identification and antimicrobial susceptibility testing**: Identification and antimicrobial susceptibility testing of the bacteria and candida were made per the instruction from VITEX^®^ 2 Compact, BioMeriéux. The technique of Kirby-Bauer disc diffusion on Mueller-Hinton medium was carried out for various antibiotics, including cefoperazone/sulbactam (105 µg), teicoplanin (30 µg), fosfomycin (50 µg), and imipenem (10 µg). The inoculum concentration to be tested was equivalent to the 0.5 McFarland standards in sterile saline. The published CLSI guideline was used to determine the susceptibility result of each antibiotic tested.^14^ Our laboratory used the following recommended organisms as the quality control, i.e., *Escherichia coli* (*E. coli*) ATCC 25922, *Pseudomonas aeruginosa* (*P. aeruginosa*) ATCC 27853, and *Staphylococcus aureus* (*S. aureus*) ATCC 25923.

**Statistical analysis**: Data attained were entered and tabulated into Excel files (Microsoft Excel, Microsoft Corp. Redmond, WA, USA). Statistical analyses were performed using Statistical Package for Social Sciences (SPSS) Statistics Version 21.0 (IBM Corp., Released 2012, Armonk, NY, USA). Descriptive analyses were used to see the frequency distribution.

## Results

During the study period, 808 isolates fulfilled the study criteria. The demographic characteristics showed that the mean age was 6.2 days (range 0–40 days) and female neonates (501 [62.0%]) dominated the study population. Of the total 808 isolates, we analyzed 36 (4.5%) sputum samples, 724 (89.6%) blood samples, 37 (4.6%) urine samples, and one (0.1%) CSF samples (Table 1). A total of 132 (16.3%) samples revealed positive culture, out of which 76 (57.6%) samples revealed MDR pathogens. The various pathogens year-by-year reported were 71 (53.8%) GNB, 51 (38.6%) GPB, and 10 (7.6%) *Candida spp.* isolates.

**Table 1:** Year-on-year general profile among isolates detected in NICU, 2011–2019

Year	2011	2012	2013	2014	2015	2016	2017	2018	2019	Total
	n=53	n=131	n=134	n=47	n=86	n=103	n=83	n=89	n=82	n=808
Age (mean, day)	4	7	7	8	4	6	5	8	7	6.22
Gender (female)	34(64.2)	86 (65.6)	78 (58.2)	23 (48.9)	54 (62.8)	73 (70.9)	48 (57.8)	55 (62.9)	50 (62.2)	501 (62.0)
Specimen source										
Sputum	4 (7.6)	9 (6.9)	8 (6.0)	3 (6.4)	1 (1.2)	4 (3.9)	5 (6.0)	2 (2.3)	-	36 (4.5)
Blood	46 (86.8)	114 (87.0)	114 (85.7)	41 (87.2)	81 (94.2)	95 (92.2)	74 (89.2)	83 (93.3)	76 (92.7)	724 (89.6)
Urin	3 (5.6)	5 (3.8)	11 (8.2)	3 (6.4)	3 (3.4)	2(1.9)	2 (2.4)	3 (3.4)	5 (6.1)	37 (4.6)
Pus and CSF	-	1 (0.7)*	1 (0.1)**	-	1 (1.2)*	1 (1.0)*	1 (1.2)*	1 (1.1)*	-	6 (0.7)
Feces	-	2 (1.5)	-	-	-	1 (1.0)	1 (1.2)	-	1 (1.2)	5 (0.6)
Positive culture	17 (32.1)	25 (19.1)	26 (19.4)	8 (17.0)	10 (11.6)	14 (13.6)	10 (12.0)	10 (11.2)	12 (14.6)	132 (16.3)
Type of pathogens (n = 132)										
GNB	9 (52.9)	12 (48.0)	16 (61.5)	4 (50.0)	5 (50.0)	9 (64.3)	6 (60.0)	7 (70.0)	3 (25.0)	71 (53.8)
GPB	8 (47.1)	10 (40.0)	8 (30.8)	1 (12.5)	5 (50.0)	5 (35.7)	4 (40.0)	3 (30.0)	7 (58.3)	51 (38.6)
*Candida spp.*	-	3 (12.0)	2 (7.7)	3 (37.5)	-	-	-	-	2 (16.7)	10 (7.6)
MDROs	11 (64.7)	13 (52.0)	18 (69.3)	3 (37.5)	4 (40.0)	7 (50.0)	5 (50.0)	6 (60.0)	9 (75.0)	76 (57.6)

Data were presented in n (%). CSF= cerebrospinal fluid; GNB= Gram negative bacteria; GNP= Gram positive bacteria; MDROs= multidrug-resistant organisms

* only pus

** only CSF

**Figure 1:**
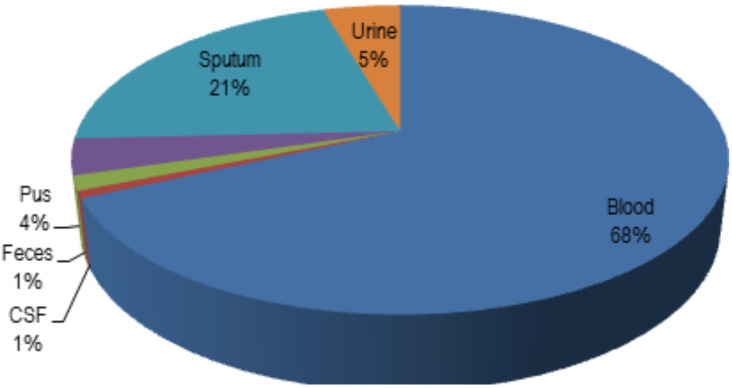
Percentage of positive culture from specimen type during study period

During the nine years of observation, there were 40 cases of coagulase-negative Staphylococci (CONS) infection; 20 acquired *Acinetobacter baumanii*, 17 *Klebsiella pneumoniae*, and nine *Enterobacter cloacae*; *P. aeruginosa* in 5 cases; *Stenotrophomonas maltophilia* and *S. aureus* in 3 of each isolates. The majority of GNB were from blood and sputum samples, and almost all of *Candida spp*. isolates were revealed from fungemia (Figure 1).

The MDR-GNB was detected in 47 (61.8%) isolates, while MDR-Ab and ESBL were revealed simultaneously in 18 (38.2%) respective isolates. There was no *Klebsiella pneumonia* carbapenemase (KPC) in this present study. The CONS accounted for 29 (38.2%) of the resistant pathogens identified from neonatal infection (Table 2). Surprisingly, positivity among male neonates' culture was found as high as 52 (16.9%) isolates and for MDR isolates 36 (69.2%) isolates were isolated. Additionally, neonates from the age group of 4–28 days revealed the maximum MDR positivity of 62 (67.4%).

**Table 2:** Distribution of multidrug-resistant organisms during study period, 2011–2019

Year	2011	2012	2013	2014	2015	2016	2017	2018	2019	Total
Type of MDROs (n= 76)										
CONS	4(5.3)	4 (5.3)	7 (9.2)	-	2(2.6)	3(3.9)	2(2.6)	1(1.3)	6(7.9)	29(38.2)
MDR-GPB	-	-	-	-	-	-	-	-	-	-
MDR-GNB	7(9.2)	9(11.8)	11(14.5)	3(3.9)	2(2.7)	4(5.3)	3(3.9)	5(6.6)	3(3.9)	47(61.8)
Type of MDR-GPB (n=0)										
CRE	-	-	-	-	-	-	-	-	-	-
MRSA/VRSA	-	-	-	-	-	-	-	-	-	-
VRE	-	-	-	-	-	-	-	-	-	-
Type of MDR-GNB (n=47)										
MDR-Ab	3 (6.4)	4 (8.5)	6 (12.8)	-	1 (2.1)	3 (6.4)	1 (2.1)	-	-	18 (38.3)
ESBL	1 (2.1)	2 (4.3)	4 (8.5)	3 (6.4)	1 (2.1)	-	2 (4.3)	3 (6.4)	2 (4.3)	18 (38.3)
KPC	-	-	-	-	-	-	-	-	-	-
Others*	3 (6.4)	3 (6.4)	1 (2.1)	-	-	1 (2.1)	-	2 (4.3)	1 (2.1)	11 (23.4)

Data were presented in n (%). MDROs= multidrug-resistant organisms; MDR-GNB= multidrug-resistant Gram negative bacteria; MDR-GPB= multidrug-resistant Gram positive bacteria; CRE=carbapenem-resistant Enterobacteriaceae; MRSA=methicillin resistant *Staphylococcus aureus*; VRSA=Vancomycin resistant *Staphylococcus aureus*; MDR-Ab=multidrug resistant Acinetobacter; ESBL=extended-spectrum beta-lactamase; KPC=*Klebsiella pneumonia* carbapenemase; CONS=coagulase negative staphylococci.

* *Pseudomonas spp, Stenotrophomonas maltophillia*, and *Sphingomonas paucimobilis*

Most of the GNB had high susceptibility against cefoperazone/sulbactam (79.6%), amikacin (88.7%), and tigecycline (77.1%). These bacteria showed moderate susceptibility to all carbapenem (imipenem and meropenem). The susceptibility to cephalosporin was generally low, except for GPB non-CONS. In contrast, most *S. aureus* dan *Streptococcus spp*. were found to have a high susceptibility against the various class of antibiotics, including vancomycin, linezolid, and teicoplanin. The susceptibility pattern of antifungal revealed excellent susceptibility (100.0%) (Table 3).

**Table 3:** Susceptibility pattern among various antimicrobial drugs.

Various antimicrobial drugs	% Susceptibility			

	GNB	GNP	CONS	*Candida spp.*
Amoxicillin/clavulanate	13.0	100.0	50.0	-
Amoxicilln	8.6	50.0	7.7	-
Ampicillin	11.9	33.3	50.0	-
Ceftazidime	42.9	85.7	-	-
Ceftriaxone	32.8	100.0	22.5	-
Cefotaxime	27.8	100.0	17.5	-
Cefepime	43.2	100.0	20.7	-
Ampicillin/sulbactam	16.7	100.0	57.1	-
Cefoperazone/sulbactam	79.6	-	-	-
Piperacillin/tazobactam	52.2	100.0	20.5	-
Fosfomycin	70.0	-	-	-
Ertapenem	93.5*	100.0	20.6	-
Imipenem	66.7	100.0	17.9	-
Meropenem	67.6	75.0	20.0	-
Tigecycline	77.1	100.0	100.0	-
Vancomycin	-	100.0	100.0	-
Linezolid	-	100.0	100.0	-
Teicoplanin	-	100.0	100.0	-
Gentamycin	30.4	85.7	57.5	-
Amikacin	88.7	66.7	-	-
Trimethoprim/sulfamethoxazole	41.3	100.0	56.4	-
Ciprofloxacin	54.2	81.8	57.5	-
Levofloxacin	69.6	88.9	57.5	-
Amphotericin B	-	-	-	100.0
Fluconazole	-	-	-	100.0
Voriconazole	-	-	-	100.0
Micafungin	-	-	-	100.0

* Not included *A. baumanii*, *P.aeruginosa* and *Stenotrophomonas maltophilia*

## Discussion

The antimicrobial resistance crisis represented a public and global problem recently. Nonetheless, antimicrobial had beneficial effects on human life, including neonatal infection (1,15). Neonatal infection is the leading cause of morbidity and contributes to approximately 30%–99% of mortality rates in developing countries (16). Among the 3.5 million cases per year in South Asia, a study in Indonesia counted a five percent incidence of neonatal sepsis with a 28.3% mortality rate (16,17). This clinical burden was found similar to our study, amongst 808 isolates, with the most common infection source came from 68.2% blood specimens. Some previous studies from other countries observed that 14.24%–44% of pathogens recovered from suspected patients with bacteremia and presented as the predominant cause among neonates admitted in NICU (2,18–21). It has been widely reported that the incidence of neonatal sepsis was associated with late-onset sepsis (56%–≥ 72.%), low and very low birth weight (50%–60%), pre-term gestational (51.04%–51%), and empirical boardspectrum antibiotics (4–5,10,21). A study conducted in a tertiary woman and child hospital in Indonesia showed no relationship between neonatal sepsis, gestational age, and prophylaxis antibiotics given during delivery (21).

The studies related to the pathogenic profile and their susceptibility among neonates were very limited in Indonesia or other regions worldwide due to most analyses were based on blood culture. A similar investigation conducted in Bali and Jakarta merely portrayed the incidence, risk factor or bacteria strain, such as *Serratia spp.* as the most prominent neonatal infection (17,21). With the declared MDR pathogens as a major threat (1,11), our observations described helpful and insightful information for clinical practice in guiding appropriate empirical antimicrobial therapy based on the pathogens distribution and susceptibility profile within an Indonesian secondary referral hospital and region with unique population characteristics. In the current setting, the prevalence of MDR-GNB was 61.8%, which was lower than the previous investigation by Gupta S et al. (67.1%) (19) or a study conducted in India by Baig K et al. (76%) (7). An investigation from Northern Taiwan found that the MDR-GNB bacteremia accounted for 18.6%, mainly ESBL-producing *K. pneumoniae* (59.6%) (5). In contrast, a study by Shah AJ et al. in a tertiary hospital revealed a 14% ESBL producer and 29% carbapenemase producer, which was quite similar to the present study (22). The striking difference between the present study and the previous studies was that no MDR-GPB such as MRSA in the bacteriological profile. Nonetheless, emerging pathogens, particularly ESBL-producing Enterobacteriaceae, MDR-*A. baumanii*, *Pseudomonas spp.*, and *Serratia marcescens* were reported as the leading cause of neonates infection in many regions (8,15,21–25).

Multiple factors related to the GNB resistance acquisition among critically ill neonates have been described (3,5). Acquisition of antibiotic-resistant GNB as the causative pathogen impacting the MDR-GNB colonization was significantly associated with 1) invasive medical devices or procedures, 2) excessive use or over-prescription of a third-generation cephalosporin, 3) the length of hospitalization in NICU, and 4) low birth weight and/or 5) gestational age (1,2–3,15). Giuffre M et al. proved that the days of exposure to ampicillin/sulbactam was related to the acquisition of ESBL-producing GNB (OR 1.040, 95% CI 1.009–1.071) (3). On the other side, it was known that this organism's attitude was spreading by cross-contamination and quickly transferring their ESBL genes, specifically ^bla^TEM/SHV and ^bla^CTX-Mtypes, which were related to cross-infection in hospitals (3,26).

Patel SJ et al. notified that low birth weight (< 1000 gram) recipients of prolonged antibiotics as well as maternal colonization in the community were highly susceptible to resistant GNB (15). Therefore, these circumstances' adverse outcome reflected a treatment failure that may lead to a two or three-fold higher morbidity or mortality rate, increased resource utilization, higher hospitalization costs, and increased use of newer broad-spectrum antibiotic therapies (1,5,27). The present study's level of resistance suggested that MDR-GNB appeared as an important pathogen of neonatal infection. Thus, focusing on appropriate antimicrobial usage and improving infection control should be implemented in NICU.

The GNB found in this investigation was resistant to various antibiotic classes, including β-lactams and β-lactam/β-lactamase inhibitor combinations and cephalosporins; nonetheless remained susceptible to carbapenems, glycylcycline (tigecycline), and an aminoglycoside (amikacin). Referring to the healthcare-associated infection (HAI) definition by the CDC's National Healthcare Safety Network in which an infection acquired by patients in a hospital on/after the third day of admission for non-infectious conditions (11,28), highlighted that most of the GNB was found to be closely associated with HAI. Previous studies reported the GNB was the preponderance of HAI, 53%, 76%, and 67.1%, respectively (2,7,19). Likewise, a review by Teerawattnapong et al. that carbapenem-resistant *Acinetobacter baumanii* (CRAB, 64.91%) and MDR-Ab (58.51%) were associated with HAI in Southeast Asia (29). The increasing threat of MDR-GNB and occasional pan-resistant among neonates are alarming for strict monitoring, infection control, and antimicrobial stewardship. Furthermore, the emerging of KPC encoding New Delhi metallo-beta-lactamase (NDM-1) or healthcare-acquired *A. baumanii* infection is worrisome since attributed to mortality, morbidity, economic burden, and the health care system (2,27).

Our pathogen profile was different from that found in some previous analyses. There was a high prevalence of GNB (53.8%) and a lower percentage of *S. aureus* or Streptococci, compared with a predominance of Grampositive cocci, especially MRSA or group beta streptococci as in other developed countries (15,30–31). The pathogen profile difference may implicate a highly and strict application of hygienic and aseptic practices in the delivery rooms and the NICU (29). The epidemiology of resistant pathogens among the NICU population mostly focused on late-onset sepsis. Hence, GBP was the more common cause of neonate infections. The CONS and *S. aureus* were the leading pathogens among GPB isolated from the hospital setting (15,18–19). Of all GPB, CONS were the majority isolates, similar to the study by Sarangi KK et al. observed *S. haemolyticus* (34.1%), *S. epidermidis* (14.6%), and followed *S. aureus* (7.3%) (4). Contrary to a tertiary hospital study in India, GPB (60.37%) was revealed as the dominant pathogen, followed by GNB (36.3%) and *Candida spp*. (3.3%) (18).

In the present study, ten (7.6%) neonates demonstrated fungal infection due to *Candida spp.* This finding was comparable to the outcome of another investigation, which showed that fungi (15.8%) predominantly *Candida albicans* (9.8%) (4). Our result was higher than the study by Banik A et al., who notified 3.33% fungemia with a predominance of *Candida non-albicans* (18). All of candida isolates remained 100% susceptible against various antifungal classes. Nevertheless, there has been an emergence of Candida non-albicans/nonparapsilosis resistant against fluconazole due to prophylaxis; fortunately, fluconazole resistance has not been detected among our isolates. Therefore, a long and strict follow up may be required to estimate the risk (15). The cause of fungal infection have been described, including 1) the overuse or inappropriate broad-spectrum antibiotic therapy, 2) prolonged hospitalization, 3) medical devices, and 4) impairment in the intestinal mucosa (18).

The outcome of our report was based on laboratory data, which was a particular limitation. Nevertheless, depending on the information of culture and Gram staining result and in conjunction with elevated white blood cells, it was concluded that a significant number of MDR-GNB were being isolated as the pathogens of neonatal infection. The low level of susceptibility to cephalosporins, β-lactams and β-lactam/β-lactamase inhibitor combinations, and even fluoroquinolones, which were not used in neonates, indicating a possible high burden of resistant pathogens as well as the prolonged use of empirical therapy. Implementation of antimicrobial stewardship in the NICU for appropriate use of antimicrobial in neonates should be done to reduce multidrug pathogens. Development of suitable antimicrobial stewardship required multidisciplinary teams, local antibiotic guidelines by NICU-specific susceptibility pattern, microbiology laboratory, and clinical knowledge to promptly discontinuing or switching antimicrobial therapy.

Our study provided much-needed information on the profile of pathogenic bacteria from various source of infection and their antimicrobial susceptibility for NICU patients in the context of global literature. Highly resistant organisms threaten the extraordinary patient's health benefit that has been achieved by various antimicrobials. This finding may reflect the overuse of these drugs and needed a review of antimicrobial guidelines and policy for better healthcare planning and clinical outcome. An evidence-based approach such as accurate and prompt diagnosis and treatment, prudent use of antibiotics, and strict prevention of transmission will effectively overcome the spread of MDROs. Future studies are substantial for mapping the distribution and development of MDROs in establishing local and global strategies as an effective control tool against infectious disease and drug resistance.
